# The Impact of Ubiquitous Face Masks and Filtering Face Piece Application During Rest, Work and Exercise on Gas Exchange, Pulmonary Function and Physical Performance: A Systematic Review with Meta-analysis

**DOI:** 10.1186/s40798-021-00388-6

**Published:** 2021-12-11

**Authors:** Tobias Engeroff, David A. Groneberg, Daniel Niederer

**Affiliations:** 1 Division Health and Performance, Institute of Occupational, Social and Environmental Medicine, Goethe University Frankfurt, Theodor-Stern-Kai 7, Building 9B, 60590 Frankfurt am Main, Germany; 2 Institute of Occupational, Social and Environmental Medicine, Goethe-University Frankfurt, Frankfurt am Main, Germany; 3 Department of Sports Medicine and Exercise Physiology, Institute of Sport Sciences, Goethe-University Frankfurt, Frankfurt am Main, Germany

**Keywords:** Corona, Crisis, Upper airway infection, Droplets, Sport, Dead space

## Abstract

**Background:**

Protection against airborne infection is currently, due to the COVID-19-associated restrictions, ubiquitously applied during public transport use, work and leisure time. Increased carbon dioxide re-inhalation and breathing resistance may result thereof and, in turn, may negatively impact metabolism and performance.

**Objectives:**

To deduce the impact of the surgical mask and filtering face piece type 2 (FFP2) or N95 respirator application on gas exchange (pulse-derived oxygen saturation (SpO_2_), carbon dioxide partial pressure (PCO_2_), carbon dioxide exhalation (VCO_2_) and oxygen uptake (VO_2_)), pulmonary function (respiratory rate and ventilation) and physical performance (heart rate HR, peak power output W_peak_).

**Methods:**

Systematic review with meta-analysis. Literature available in Medline/Pubmed, the Cochrane Library and the Web of Knowledge with the last search on the 6^th^ of May 2021. Eligibility criteria: Randomised controlled parallel group or crossover trials (RCT), full-text availability, comparison of the acute effects of ≥ 1 intervention (surgical mask or FFP2/N95 application) to a control/comparator condition (i.e. no mask wearing). Participants were required to be healthy humans and > 16 years of age without conditions or illnesses influencing pulmonary function or metabolism. Risk of bias was rated using the crossover extension of the Cochrane risk of bias assessment tool II. Standardised mean differences (SMD, Hedges' g) with 95% confidence intervals (CI) were calculated, overall and for subgroups based on mask and exercise type, as pooled effect size estimators in our random-effects meta-analysis.

**Results:**

Of the 1499 records retrieved, 14 RCTs (all crossover trials, high risk of bias) with 25 independent intervention arms (effect sizes per outcome) on 246 participants were included. Masks led to a decrease in SpO_2_ during vigorous intensity exercise (6 effect sizes; SMD = − 0.40 [95% CI: − 0.70, − 0.09], mostly attributed to FFP2/N95) and to a SpO_2_-increase during rest (5 effect sizes; SMD = 0.34 [95% CI: 0.04, 0.64]); no general effect of mask wearing on SpO_2_ occurred (21 effect sizes, SMD = 0.34 [95% CI: 0.04, 0.64]). Wearing a mask led to a general oxygen uptake decrease (5 effect sizes, SMD = − 0.44 [95% CI: − 0.75, − 0.14]), to slower respiratory rates (15 effect sizes, SMD = − 0.25 [95% CI: − 0.44, − 0.06]) and to a decreased ventilation (11 effect sizes, SMD = − 0.43 [95% CI: − 0.74, − 0.12]). Heart rate (25 effect sizes; SMD = 0.05 [95% CI: − 0.09, 0.19]), W_peak_ (9 effect sizes; SMD = − 0.12 [95% CI: − 0.39, 0.15]), PCO_2_ (11 effect sizes; SMD = 0.07 [95% CI: − 0.14, 0.29]) and VCO_2_ (4 effect sizes, SMD = − 0.30 [95% CI: − 0.71, 0.10]) were not different to the control, either in total or dependent on mask type or physical activity status.

**Conclusion:**

The number of crossover-RCT studies was low and the designs displayed a high risk of bias. The within-mask- and -intensity-homogeneous effects on gas exchange kinetics indicated larger detrimental effects during exhausting physical activities. Pulse-derived oxygen saturation was increased during rest when a mask was applied, whereas wearing a mask during exhausting exercise led to decreased oxygen saturation. Breathing frequency and ventilation adaptations were not related to exercise intensity. FFP2/N95 and, to a lesser extent, surgical mask application negatively impacted the capacity for gas exchange and pulmonary function but not the peak physical performance.

*Registration*: Prospero registration number: CRD42021244634

## Key Points

- The application of masks (filtering face pieces type 2, N95 respirators and surgical face masks) tends to increase pulse-derived oxygen saturation during rest, whereas oxygen saturation during graded exercise until volitional exhaustion tends to decrease if a mask is applied.
- The application of masks alters respiratory rate and ventilation.
- Compared to surgical face masks, filtering face pieces type 2 and N95 respirators have a greater impact on gas exchange.
- Alterations in pulmonary function and gas exchange during mask wearing at rest are different to the effects of mask wearing during physical activity.
- Mask application during exhausting activities showed the greatest impact on oxygen uptake.

## Background

During the ongoing COVID-19 pandemic, the application of mouth and nose protection against droplets and aerosols has drastically increased. In particular, during public transport use, work and leisure time, mouth and nose protections are ubiquitous. The World Health Organization recommends to wear such a mouth and nose protection mask in public settings if a physical distance ≥ 1 m cannot be ensured [1]. This recommendation also includes outdoor settings.

Although not specifically recommended to suppress transmission in public settings, current evidence suggests that surgical masks and filtering face pieces type 2 or N95 respirators (FFP2 have comparable features to N95 respirators) are more effective in filtering particle emission compared to cloth masks [2]. A direct comparison between these two medical type masks (FFP2/N95 and surgical masks) revealed no significant differences in the effectiveness against influenza [3] which led to the assumption that both may also be suited to reduce the risk of other airborne infections. Consequently, FFP2/N95 and surgical mask wearing is currently (November 2021) recommended in pandemic circumstances, such as the current COVID-19-crisis, during rest and light to moderate physical activities, but also during physical labour and other indoor activities with vigorous intensity.

Except for the recommendation to exercise outside with social distancing to avoid a potential risk for reduced breathing capacity, the WHO currently does not limit the application of face masks to healthy individuals [1]. However, two different mask and respirator related adaptations are currently considered to affect gas exchange during rest and exercise. Both medical mask types include multiple layers and materials and, thus, based on this construction, it is likely that increased breathing resistance affects respiration during rest and exercise [4]. Since the detrimental effects of breathing resistance are associated with exercise intensity [5], decreased ventilation and tidal volumes might limit oxygen uptake and carbon dioxide exhalation, especially during strenuous physical activities.

Depending on the fit of the mask to the individual’s face, it is also possible that exhaled air is trapped within the space between the face and the device. Consequently, this proportion of inspired air is rebreathed and may contain higher concentrations of carbon dioxide and lower oxygen compared to ambient air [6]. Since tidal volume and respiratory rate increase during exercise [7], it is likely that the impact of this small portion of trapped exhaled air is inversely associated with the intensity of physical activities; higher intensities are suggested to lead to lower effects.

Surgical masks are applied as a barrier to reduce the direct transmission of infectious liquids or aerosols from the wearer and also to avoid contact with droplets [8]. Filtering face pieces are tighter fitting in order to meet specific requirements for the filtration of small airborne particles [8].

Both adaptations (breathing resistance and exhaled air rebreathing) might affect gas exchange more severely when an FFP2/N95 is applied rather than a surgical mask. Although it is likely that the aforementioned effects may not lead to clinically relevant hypoxia or hypercapnia, the slightly elevated CO_2_ may still affect cognitive performance and could increase the risk for headache [9]. An O_2_ concentration lowering of 5%, with a concurrently decreased oxygen uptake capacity, results in increased anaerobic metabolism and lactic acid accumulation [10]. These mechanisms may limit both endurance and maximal performance [10]. Furthermore, increased breathing resistance alone seems to be associated with respiratory fatigue, impaired physical work capacity and early exhaustion even at lighter workloads [4].

Some randomised controlled studies have already compared the impact of wearing a face mask during rest and physical activity [11, 12]. In contrast, only one systematic review with meta-analysis exists so far on this topic which is focussed solely on the effects during structured exercise [13]. In line with our assumptions, these authors described detrimental effects on end-tidal CO_2_, heart rate and respiratory rate and a larger impact of FFP2/N95 masks compared to surgical masks [13]. Despite the effects of FFP2/N95 and surgical masks, these authors concluded that the mask types investigated by their review can be worn during exercise with no influences on performance and minimal impacts on physiological variables [13]. These results may, however, be limited by the severely biased quality of the studies included in their review, such as non-randomised design, fixed trial orders and repetitive measures without sufficient wash-out phases [14, 15]. Beyond the need of a subgrouped analysis (grouped by mask type and differentiated according to the impact during rest and physical activity with different intensities), future systematic reviews on randomised controlled trials (RCT) are necessary in order to investigate the potential detrimental effects of surgical mask and FFP2/N95 respirator application during settings relevant for everyday life, including rest and different states of physical activity.

The objectives of this systematic review with meta-analysis were to compare the impact of FFP2/N95 respirators and surgical face mask application to each other and to wearing no mask. The outcomes of interest were pulmonary function (respiratory rate, ventilation- and tidal volumes) and markers of gas exchange (oxygen saturation, carbon dioxide partial pressure, carbon dioxide exhalation and oxygen uptake) during rest and physical activity with low, moderate and vigorous intensities. A secondary goal was to analyse a potentially detrimental impact on physical performance during exhausting activities (heart rate, peak power output).

We hypothesised, by considering oxygen uptake and carbon dioxide exhalation, that (1) a general effect of mask wearing occurs, (2) that the FFP2/N95 mask leads to a larger decrease in oxygen uptake and carbon dioxide exhalation capacity when compared to surgical masks and no mask wearing and that (3) this effect is more pronounced during exhausting exercises.

## Methods

### Study Design

This secondary data analysis was conducted as a systematic review with meta-analyses and meta-regression. The Preferred Reporting Items for Systematic Reviews and Meta-Analyses (PRISMA) guidelines [16] were applied. The review was preregistered in the PROSPERO database (CRD42021244634). The date of submission was 23.03.2021, with the registration on 24.03.2021. An update was registered on 01.06.2021.

### Inclusion and Exclusion Criteria

Studies on healthy (asymptomatic) participants (over the age of 16) were searched for. To be included in the review, the study had to investigate the effects of at least one frequently applied medical face-nose-mask (N95, FFP2, and/or surgery mask) in a controlled design (control/comparator arm: no mask). Outcomes of interest were (1) metabolic measures including oxygen, carbon dioxide and heart rate via cardiopulmonary exercise testing (CPET) and/or invasive (arterial, venous or capillary) blood gas analysis and/or transcutaneous oximetry and potentiometry or (2) spirometry measures indicating breathing effort (respiratory rate and ventilation). Further inclusion criteria were for the study to be an original data publication adopting a randomised controlled design (crossover or parallel group) and an accessible abstract in English.

Exclusion criteria included studies having participants suffering from non-common conditions or who had specific sample characteristics such as obesity or pregnancy. Further exclusion criteria were medication and diseases potentially affecting respiratory outcomes such as cardiopulmonary disease (e.g. chronic obstructive pulmonary disease or heart failure), cancer, infection, inflammatory arthropathy, bleeding disorders (e.g. haemophilia), spinal disease (e.g. herniation of the lumbar disc), high-velocity trauma or fracture and the presence of severe or progressive neurological deficits.

### Literature Research

The literature search was performed between February and May 2021. The final search date was up to and including 05 May 2021. The search was performed in PubMed (Medline), Web of Knowledge and the Cochrane Library/Cochrane Central Register of Controlled Trials (CENTRAL, with EMBASE) without publication language restrictions. In addition, hand searching Google Scholar to find potential grey literature was performed.

We applied a search strategy including terms for physical activity, occupational activities and exercise, different mask and face piece types and the Covid pandemic. Furthermore, we applied hand searching in the reference citations of previously identified articles (cross-referencing). Potentially relevant articles were searched for adopting the following Boolean search syntax (example for the PubMed search): (“Mask” OR “Facemask” OR “Filtering Face Piece” OR “FFP2” OR “N95” OR “N99” OR “respirator”) AND (“oxygen” OR “carbon dioxide” OR “metabolic” OR “blood gas” OR “hypoxia” OR “hypoxic” OR “hypercapnia” OR “hypercapnic” OR “CO2” OR “O2” OR “aerobic”) NOT (anaesthesia OR laryngeal OR nasal). An initial exploratory electronic database search was independently conducted by two reviewers (TE and DN) to define the final search terms and operators. The same reviewers performed the main search. Studies identified through the search strategy were screened for between-database duplicates before the abstract screening. Subsequently, both reviewers independently screened the identified studies, in duplicate, to determine whether they met the inclusion criteria. The herewith identified studies were screened for eligibility using (1) titles and (2) abstracts. The remaining full texts were assessed to ascertain whether they fulfilled the inclusion criteria whilst not fulfilling the exclusion criteria. Differences in opinion relating to inclusion and exclusion were discussed until a consensus was reached. Persisting disagreements were discussed in a consensus meeting of all three authors to make the final decision. In the included studies, a citation searching was undertaken to find potential further sources.

### Data Extraction

We extracted the following descriptive information from the included studies: authors and year of publication, study design, sample size, participant characteristics, interventions, measured outcomes and major findings (outcomes not included in the meta-analysis); for this purpose, a data extraction form (Excel spreadsheet) was used. One researcher recorded all the pertinent data from the included articles and the other author independently reviewed the extracted data for their relevance, accuracy and comprehensiveness. A consensus was used to address any disparities where a third reviewer (DG) was asked, if necessary, to address the disparities. Authors of the studies included in this review who had not reported sufficient details in the published manuscript were personally addressed via email for the provision of further data. The primary outcome of the meta-analysis was pulse-derived oxygen saturation. If a study assessed more than one outcome, all data (i.e. means and standard deviations) needed to calculate the effect sizes (ES) were extracted. Missing data (means, standard deviations) were imputed from medians, interquartile range, figures and/or confidence intervals using standard procedures [17]. All studies included were screened for common effect estimators (for oxygen, carbon dioxide, heart rate and breathing data) to be included in the quantitative analysis.

### Risk of Bias

Two reviewers (TE and DN) rated the risk of bias of the included studies using the Revised Cochrane risk of bias tool (RoB II) extension for randomised crossover trials. The outcomes were graded for risk of bias in each of the following domains: sequence generation, allocation concealment, differences in baseline values, number of participants, period and carryover effects, blinding (participants, personnel and outcome assessment), incomplete outcome data, selective outcome reporting and other sources of bias. Each item was rated as having a “high risk”, “low risk” or “unclear risk” of bias and disagreements were discussed between the raters. If a decision could not be reached after discussion, a third reviewer (DG) was included to resolve any conflicts. If applicable, the outcomes’ biases were reported pooled for studies. The risk of bias findings were displayed using a traffic light system and summary plots made via an online tool created on the R package robvis [18].

The risk of bias across the studies was displayed by using funnel plots/graphs (primary outcome only). The R-based program jamovi (The jamovi project (2021), jamovi (Version 1.0.7.0) retrieved from https://www.jamovi.org; Sydney, Australia) was used for funnel plotting.

### Quantitative Analyses of Main Treatment Effects

Weighted (standardised in cases of non-unique assessment devices) mean differences (Hedges' g) were used for data pooling. A restricted maximum-likelihood random-effects meta-analysis model for continuous outcomes was chosen. For variance description, 95% confidence intervals were calculated and the summary estimates of the data were displayed using forest plots (mean effect sizes and 95% confidence intervals): (1) overall (main) effects (of mask wearing on the respective outcome) and (2) quantitative subgroup analyses. The subgrouping variables were the different mask types (surgery mask and FFP2/N95 with and without valve) and the different types of physical activity (rest, low-, moderate- and vigorous intensity). The main effects (masks and physical activity intensity) and interactions were calculated. For all effect calculations, mask wearing group effects were calculated in comparison with the comparator/control no mask wearing as standardised mean differences. To test for overall effects, Z-statistics at a 5% alpha-error-probability level were calculated for all quantitative comparisons. Clinical heterogeneity between the study's results in effect measures was assessed using I^2^- and Tau^2^-statistics. All main treatment effects analyses were performed using the MAJO package in jamovi (Version 1.0.7.0).

### Sensitivity Meta-regression Analysis

A sensitivity meta-regression on the impact of independent variables (age, mask type and exercise intensity) on the primary outcome of pulse-derived oxygen saturation was performed. A syntax for SPSS (IBM SPSS 25; IBM, USA) was used (David B. Wilson; Meta-Analysis Modified Weighted Multiple Regression; MATRIX procedure Version 2005.05.23). Inverse variance weighted regression models with random intercepts (random-effects model, fixed slopes model) were calculated. Homogeneity analysis (Q and p values), meta-regression estimates (95% confidence intervals and p values) and Z-statistics were calculated.

## Results

### Study Selection

**Fig. 1:**
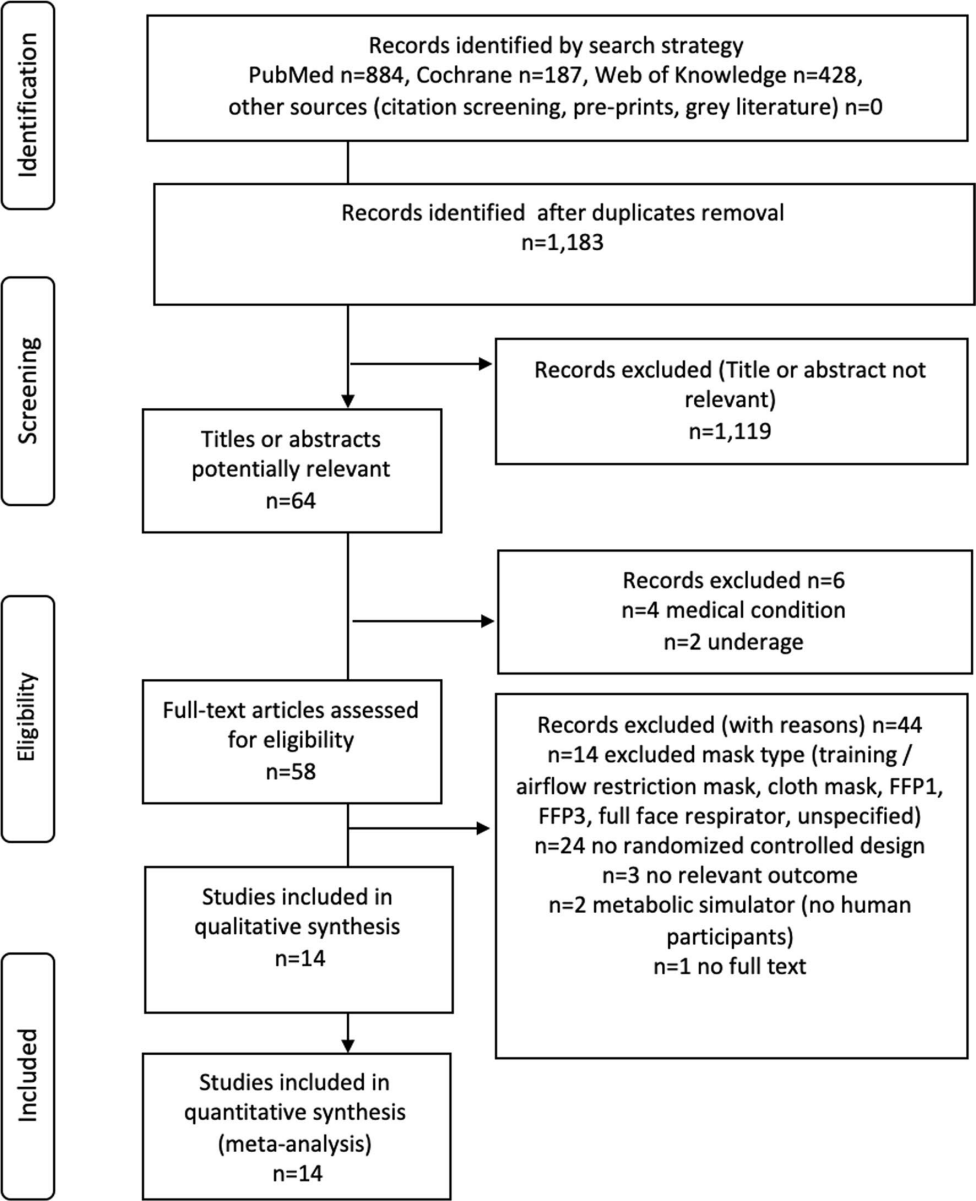
Research, selection and synthesis of included studies. n, number; FFP, filtering face piece

The review yielded 1183 unique records. After applying inclusion and exclusion criteria, 14 randomised controlled trials were included in the qualitative and quantitative analyses. Figure 1 outlines the research procedure and the flow of the study selection and inclusion.

### Results and Characteristics of Individual Studies

All 14 included studies were randomised controlled trials with a crossover design and compared one or multiple surgical masks or filtering face pieces (FFP2/N95) with, or without, exhalation valves against a control intervention without wearing a mask. The results of the individual studies (methodological aspects, participant characteristics), with a focus on the descriptive summary statistics for each group of the included studies, are displayed in Table 1. Overall, 246 participants were included.

**Table 1:** Study characteristics of the included randomised controlled crossover studies

References	Participants, number (n) (age in years, height in centimetres, weight in kilogrammes) [mean, SD]	Protocol (duration in minutes, intensity categorisation)	Mask types (surgical mask or FFP2/N95 type including manufacturer details)	Pulse-derived oxygen saturation in per cent	Heart rate in beats per minute	Transcutaneous carbon dioxide partial pressure in millimetres of mercury	Respiratory rate in breaths per minute	Ventilation in litres per minute	Oxygen uptake in millilitres per minute per kilogramme body weight	Carbon dioxide exhalation in millilitres per minute	Peak power in Watts	Secondary outcomes
				Mean, standard deviation of mask condition (no mask control)								
Roberge et al. 2014 [12]	*n* = 22 (women only) age = 26.1, 4 height = 167.5, 5.9 weight = 67.5, 9.5	Sitting (20 min; rest)	FFP2/N95 respirator without valve (flat folded or premould randomised)	99.2, 0.7 (99.0, 0.7)	73.7, 14.8 (70.6, 12.8)	36.8, 3.1 (36.9, 3.0)	17.3, 3.2 (17.8, 3.8)					Chest wall temperature, aural temperature
		Standing upright (20 min; rest)		99.1, 0.6 (98.7, 1.6)	78.6, 22.2 (76.9, 14.8)	35.1, 8.4 (37.4, 4.6)	17.2, 3.1 (17.3, 3.6)					
		Cycle ergometer (20 min; low intensity)		98.8, 0.7 (98.7, 1.2)	105.5, 15.9 (98.8, 18.2)	38.7, 3.1 (37.4, 3.3)	24.9, 6.1 (26.4, 4.2)					
Li et al. 2021 [26]	*n* = 5 (women only) age = 21.1, 0.5 height = 157.0, 3.8 weight = 49.1, 2.5	Cycle ergometer (Duration n.a.; graded until volitional exhaustion; vigorous intensity)	Surgical mask		162.6, 21.7 (162.6, 17.7)			43.2, 11.7 (51.8, 13.4)	23.0, 5.5 (26.9, 4.9)		195.6, 30.0 (197.6, 29.3)	Rating of perceived exertion (RPE)
	*n* = 5 (men only) age = 21.0, 1.6 height = 172.6 ± 4.5 weight = 59.4, 3.5				170.6, 11.3 (177.6, 10.5)			62.6, 15.4 (74.0, 13.4)	28.5, 4.9 (36.2, 3.8)		184.8, 26.8 (187.0, 28.15)	
Kienbacher et al. 2021 [21]	*n* = 48 (8 women); age = 28 ± 8; weight = n.a	Basic life support (12 min; moderate intensity)	FFP2/N95 respirator without valve (Yao Wang Medical Protective Face Maskî, QingzhouYaowang Pharmaceuticals Co. Ltd., China);	96.0, 2.7 (97.0, 2.7)	110.0, 51.3 (105.0, 51.3)							Basic life support performance; blood pressure; RPE, end-tidal CO_2_
			FFP2/N95 respirator with valve (Meditrade Respima1 EECî, Meditrade1 GmbH, Germany)	97.0, 2.7 (97.0, 2.7)	108.0, 51.3 (105.0 51.3)							
Fikenzer et al. 2020 [8]	*n* = 14 (men only); age = 38.1 ± 6.2; height = 183.0 ± 7.7; weight = 81.8 ± 8.4	Cycle ergometer semi recumbent (16.8 ± 2.8; graded until volitional exhaustion; vigorous intensity)	Surgical mask (SuavelÆ Protec Plus, Meditrade, Kiefersfelden, Germany);		183.0, 9.2 (187.0, 8.3)		39.3, 6.2 (40.9, 5.1)	114.0, 23.3 (131.0, 27.8)	37.9, 6.0 (39.7, 5.8)	269, 45 (277, 46)	269, 45 (277, 46)	Body plethysmography, spiroergometric and haemodynamic parameters, subjective complaints
			FFP2/N95 respirator without valve (ShaoguanTaijie Protection Technology Co., Ltd., Gao Jie, China)		182.0, 11.2 (187.0, 8.3)		36.8, 5.9 (40.9, 5.1)	98.8, 18.6 (131.0, 27.8)	34.5, 5.3 (39.7, 5.8)	263, 42 (277, 46)	263, 42 (277, 46)	
Kim et al. 2016 [22]	*n* = 12 (men only); age = 23.5 ± 1.6; height = 181 ± 6; weight = 81.8 ± 8.1	Treadmill walking (60 min; moderate intensity; 35 °C and 50% humidity)	FFP2/N95 respirator without valve (3 M model 1870 FFP2/N95 respirator FFR, 3 M, St Paul, MN)	97.8, 0.6 (97.9, 1.1)	105.9, 11.9 (106.2, 14.8)	41.3, 2.4 (40.9, 2.4)	28.4, 3.2 (28.1, 7.1)					Skin temperature, heat perception
Lässing et al. 2020 [25]l	*n* = 14 (men only); age = 25.7 ± 3.5; height = 183.8 ± 8.4; weight = 83.6 ± 8.4	Cycle ergometer (30 min, vigorous intensity)	Surgical mask (Suavel Protec Plus, Meditrade, Kiefersfelden, Germany)	95.3, 0.8 (95.2, 0.7)	160.1, 11.2 (154.4, 11.4)		32.1, 5.4 (34.0, 7.3)	77.1, 9.3 (82.4, 10.6)	33.1, 5.0 (34.5, 5.8)	2575, 310 (2685, 278)		Body plethysmography, spiroergometric and haemodynamic parameters
Sammito et al. 2021 [20]	*n* = 14 (3 women); age = 41 ± 12; height = 179 ± 11; weight = 80.7 ± 18.9	Sitting (5 min, rest)	FFP2/N95 respirator without valve (Gitemk FFP2 protective mask, Zhejiang Yinghua Technology, China)	97.4, 1.1 (96.7, 2.1)	76.0, 9.5 (76.9, 6.3)							None
Epstein et al. 2020 [24]	*n* = 16 (men only); age = 34 ± 4; height = 179 ± 7, weight = 76.3 ± 11.8	Cycle ergometer (16.3 ± 3.7; graded until volitional exhaustion; vigorous intensity)	FFP2/N95 respirator without valve (Duckbill style fluid shield 2 FFP2/N95 respirator particulate filter respirator, Halyard)	97.6, 1.3 (98.1, 1.1)	168.8, 12.8 (170.5, 11.7)		36.6, 5.4 (36.7, 7.5)				154, 30 (157, 31)	Rating of perceived exertion (Borg), blood pressure
			Surgical mask (Kimberly-Clark)	97.7, 1.6 (98.1, 1.1)	165.8, 16.0 (170.5, 11.7)		36.2, 7.0 (36.7, 7.5)				153, 31 (157, 31)	
Mapelli et al. 2021 [27]	*n* = 12 (6 women); age = 40.8 ± 12.4	Cycle ergometer (duration n.a.; graded until volitional exhaustion; vigorous intensity)	FFP2/N95 respirator without valve (KFFP2/N95 respirator particulate respirator, BYD care, China)	95.1, 3.1 (97.3, 1.2)	167.0, 16.1 (170.0, 14.0)		37.1, 4.5 (41.5, 8.0)	71.6, 21.2 (92.3, 26.0)	28.2, 8.8 (31.0, 6.7)	2268, 794 (2578, 763)	184, 54 (194, 57)	Spiroergometric data
			Surgical mask (disposable medical mask, Aiminde, China)	96.5, 1.2 (97.3, 1.2)	168.0, 16.0 (170.0, 14.0)		37.7, 5.5 (41.5, 8.0)	76.2, 21.6 (92.3, 26.0)	27.5, 6.9 (31.0, 6.7)	2217, 691 (2578, 763)	187, 54 (194, 57)	
Bertoli et al. 2021 [19]	*n* = 10 (7 women); age = 43 ± 10; height = 161 ± 5; weight = 65.7 ± 17.2	Lying down (no mask = 11.4 ± 2.7 min, mask 11.9 ± 2.2 min; rest)	FFP2/N95 respirator without valve (manufacturer details n.a.)						3.0, 0.6 (2.9, 0.5)	182, 37 (179, 34)		Energy expenditure
Person et al. 2018 [23]	*n* = 44 (26 women); age = 21.6 ± 2.8; height = 172.1 ± 8.3; 66.0 ± 11.4	Walking (6 min; moderate intensity)	surgical mask (manufacturer details n.a	98.3, 1.1 (98.3, 1.0)	134.6, 22.9 (138.7, 23.6)							
Kim et al. 2015 [11]	*n* = 16 (women only); age = 24.8 ± 3.5; height = 167.9 ± 6.3; weight = 66.0 ± 8.6	Standing upright (20 min, rest)	FFP2/N95 respirator without valve (3 M 9210 flat-fold model (3 M, St. Paul, MN) or a Moldex cup-shaped model in either medium/large size (Moldex2200) or small size (Moldex 2201) (Moldex, Culver City, CA))	99.1, 0.5 (98.5, 1.8)	78.9, 15.5 (78.1, 15.6)	37.4, 3.5 (38.1, 5.1)						Rating of perceived exertion, blood pressure
		Cycle ergometer semirecumbent (20 min, low intensity)		98.7, 0.6 (98.5, 1.3)	101.9, 15.0 (97.1, 19.3)	39.3, 3.2 (38.1, 3.4)						
		Sitting (20 min, rest)		99.1, 0.6 (98.9, 0.7)	73.5, 10.6 (67.8, 12.5)	37.4, 3.0 (37.3, 3.3)						
Shaw et al. 2020 [28]	*n* = 14 (7 women); age = 28.2 ± 8.7; height = 180 ± 5; weight = 86 ± 12	Cycle ergometer (10.3 ± 2.35 min; graded until volitional exhaustion; vigorous intensity)	Surgical mask CRC-Elm 5, Hawketree Solutions, Ottawa, ON, Canada	96.0, 3,0 (96.0, 4.0)	179.0, 19.0 (179.0, 16.0)						241, 57 (234, 56)	Continuous-wave near-infrared spectroscopy (vastus lateralis right); rating of perceived exertion
Roberge et al. 2010 [30]	*n* = 10 (7 women); age = 25.1 ± 7.2; height = 169 ± 12; weight = 76 ± 24	Treadmill walking (60 min, low intensity)	FFP2/N95 respirator without valve (cup shaped, manufacturer details n.a.);	98.1, 0.9 (98.5, 0.8)	98.1, 8.5 (92.3, 8.2)	39.7, 6.0 (40.7, 3.5)	25.2, 4.0 (27.7, 7.1)	23.4, 6.7 (20.9, 8.2)				Mask dead space gases, subjective complaints
			FFP2/N95 respirator with exhalation valve (cup shaped, manufacturer details n.a.)	98.4, 1.0 (98.5, 0.8)	95.1, 9.7 (92.3, 8.2)	41.5, 4.9 (40.7, 3.5)	25.2, 6.1 (27.7, 7.1)	21.2, 4.5 (20.9, 8.2)				
		Treadmill walking (60 min, moderate intensity)	FFP2/N95 respirator without valve	98.4, 0.7 (98.5, 0.8)	106.4, 9.2 (101.3, 11.8)	42.0, 5.6 (40.8, 3.2)	26.6, 6.8 (27.7, 8.6)	24.4, 6.0 (23.0, 6.5)				
			FFP2/N95 respirator with exhalation valve	98.2, 1.0 (98.5, 0.8)	106.4, 9.3 (101.3, 11.8)	42.6, 6.2 (40.8, 3.2)	25.5, 5.7 (27.7, 8.6)	23.0, 5.9 (23.0, 6.5)				

Information on participants’ characteristics, intervention protocols and applied mask types, as well as descriptive key results and information on secondary outcomes not included, are displayed

Seven studies analysed the effects of surgical masks, whereas 10 studies measured the impact of FFP2/N95 masks with exhalation valves (*n* = 2) and without valves (*n* = 10).

Regarding the experimental setup, four designs studied the impact of mask wearing during rest [11, 12, 19, 20], whilst twelve studies applied physical activity at low (*n* = 3) [6, 11, 12], moderate (*n* = 4) [6, 21–23] or vigorous intensities (*n* = 6) [8, 24–28].

The most common outcomes were heart rate (*n* = 13 studies), assessed via ECG or transcutaneous monitoring [6, 8, 11, 12, 20–28] and oxygen saturation (*n* = 11), measured via a pulse oximeter [6, 11, 12, 20–25, 27, 28]. Breathing frequency was assessed in seven trials [6, 8, 12, 22, 24, 25, 27] and ventilation in five [6, 8, 24–27]. Breathing gas analysis of oxygen uptake was assessed in five studies [8, 19, 25–27] and carbon dioxide exhalation was analysed by three studies (VCO_2_) [19, 25, 27]. Carbon dioxide partial pressure was measured via transcutaneous potentiometry (TcpCO_2_) by three studies [6, 12, 22]. Two studies additionally applied capillary blood sampling to assess pO_2_ and pCO_2_ [8, 20]. One of these studies also measured pH [8]. The latter three outcomes were not included in the meta-analysis due to the small number of studies involved.

### Oxygen Uptake and Saturation

**Fig. 2:**
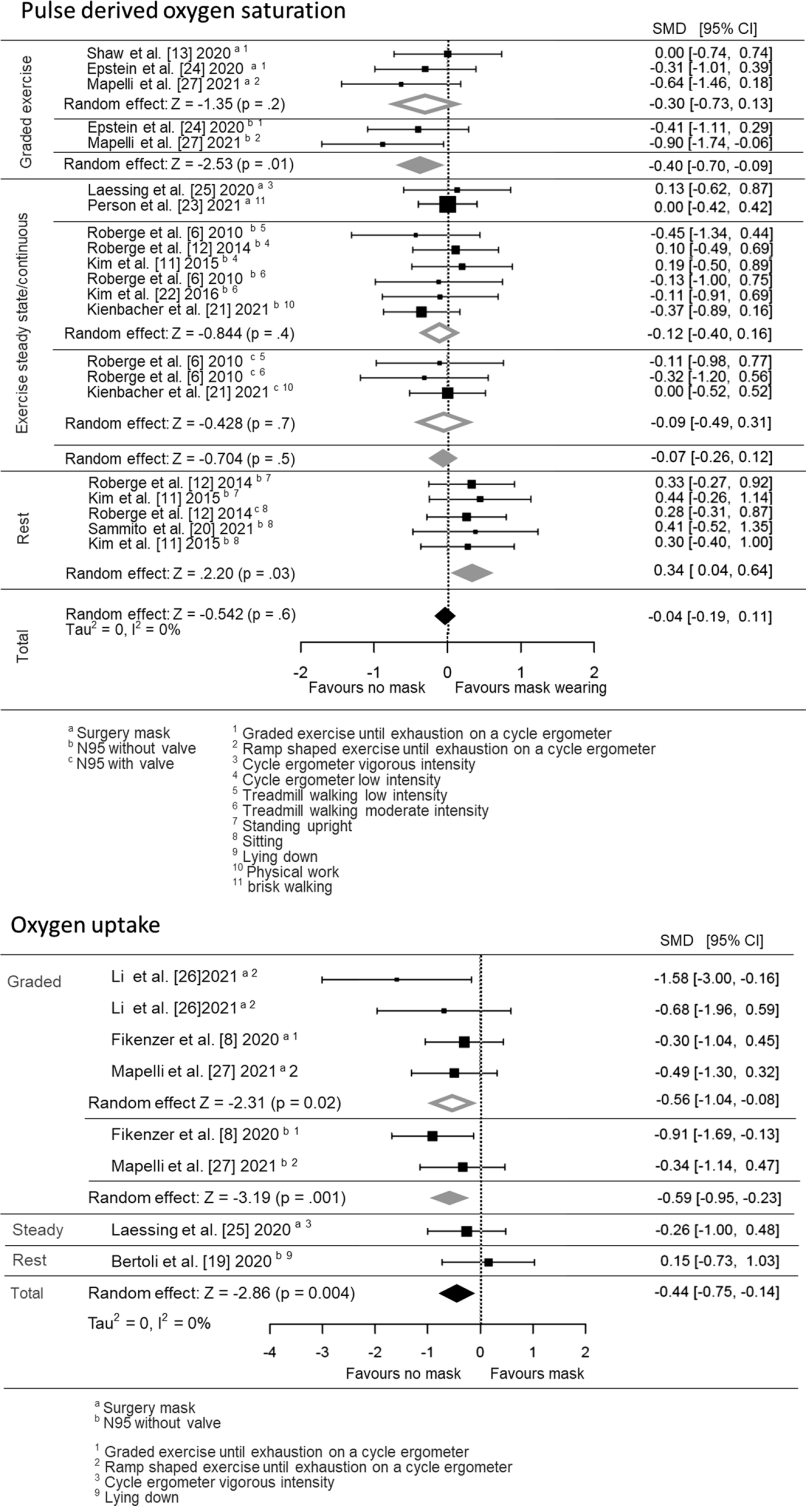
Pooled effect size estimates (standardised mean differences) for the pulse-derived oxygen saturation and oxygen uptake outcomes. Overall effects for face mask application (surgery mask and FFP2/N95 with and without valve) in comparison with a comparator/no mask control are displayed. Effects for the subgroups are based on the grouping variables of different mask types (surgery mask or FFP2/N95 with and without valve) and the different types of physical activity (rest, low-, moderate- and vigorous intensity). SMD, standardised mean difference; CI, confidence interval

The effect estimates for oxygen data are displayed in Fig. 2; both the main and subgrouped (for exercise intensity and mask type) effects are included.

Mouth and nose protection leads to a decrease in SpO_2_ during vigorous intensity exercise (6 effect sizes; SMD = − 0.40 [95% CI: − 0.70, − 0.09], mostly attributed to FFP2/N95) and to a SpO_2_-increase during rest (5 effect sizes; SMD = 0.34 [95% CI: 0.04, 0.64]) in comparison with no mask wearing. Based on these contradictory effects, no general effect of mask wearing on oxygen saturation occurred (21 effect sizes, SMD = 0.34 [95% CI: 0.04, 0.64]). Wearing surgical masks or FFP2/N95 led to a general decrease in oxygen uptake when compared to no mask wearing (8 effect sizes, SMD = − 0.44 [95% CI: − 0.75, − 0.14]). This effect occurred during exercise until volitional exhaustion when wearing either FFP2/N95 (2 effect sizes, SMD = − 0.59 [95% CI: − 0.95, − 0.23]) or surgical masks (4 effect sizes, SMD = − 0.56 [95% CI: − 1.04, − 0.08]. The two studies assessing oxygen partial pressure via invasive capillary blood gas analysis reported no effects of wearing the FFP2/N95 during rest [20] and vigorous exercise [8].

### Carbon Dioxide Exhalation and Partial Pressure

**Fig. 3:**
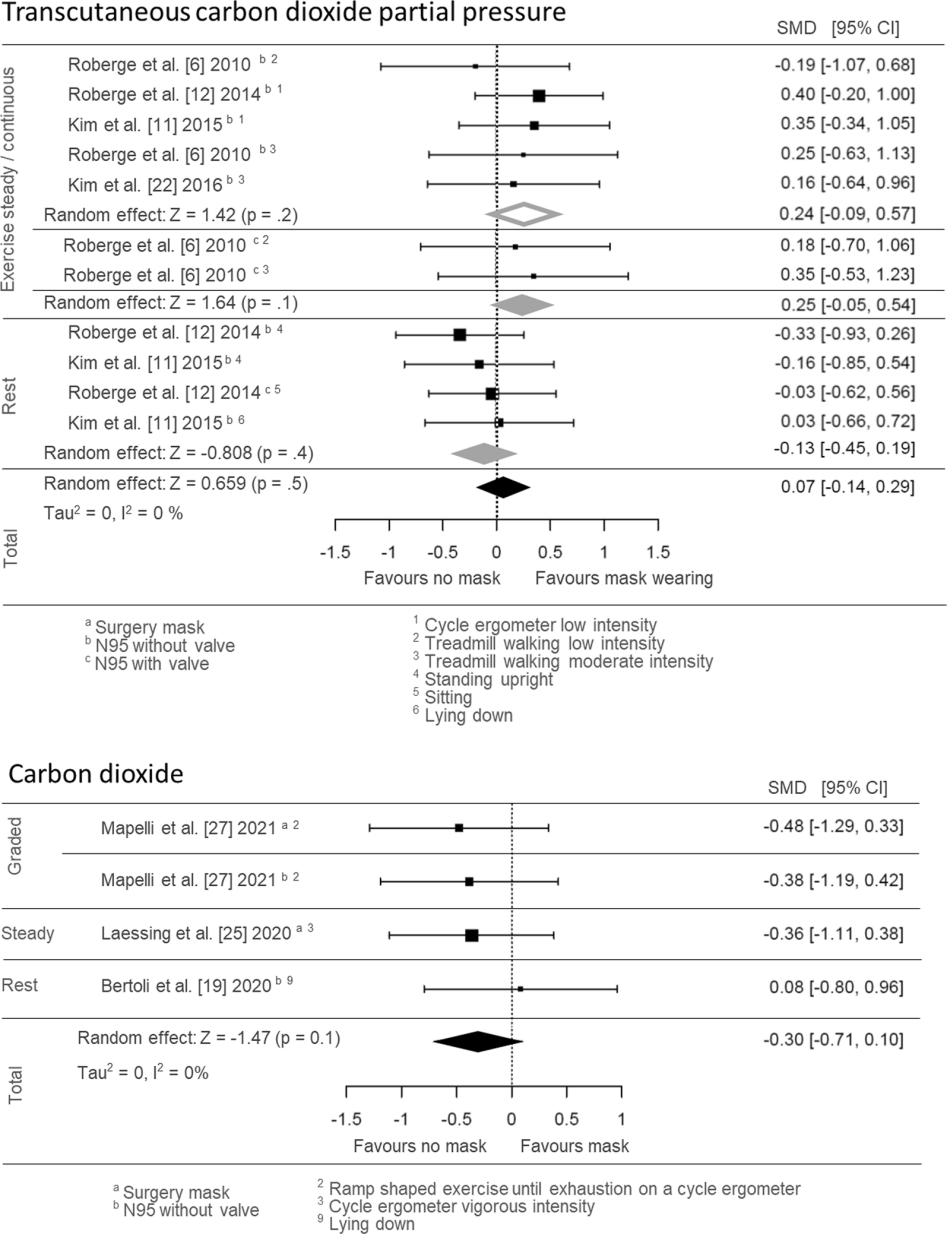
Pooled effect size estimates (standardised mean differences) for the transcutaneous carbon dioxide partial pressure and carbon dioxide exhalation outcomes. Overall effects for face mask application (surgery mask, FFP2/N95 with and without valve) in comparison with a comparator/no mask control are displayed. Subgroups were based on the grouping variables of different mask types (surgery mask, FFP2/N95 with and without valve) and the different types of physical activity (rest, low-, moderate- and vigorous intensity). SMD, standardised mean difference; CI, confidence interval

The pooled effect estimates for carbon dioxide data are displayed as forest plots in Fig. 3. Both main and subgrouped (for exercise intensity and mask type) effect estimates are shown. Carbon dioxide partial pressure (11 effect sizes; SMD = 0.07 [95% CI: − 0.14, 0.29]) and VCO_2_ (4 effect sizes, SMD = − 0.30 [95% CI: − 0.71, 0.10]) did not differ between mask and no mask wearing, neither in total nor dependent on the mask type or rest or exercise intensity (Fig. 3). As with the oxygen measures, two studies assessed carbon dioxide partial pressure via invasive blood gas analysis and, again, reported no effects of the FFP2/N95 during rest [20] and vigorous exercise [8].

### Pulmonary Function

**Fig. 4:**
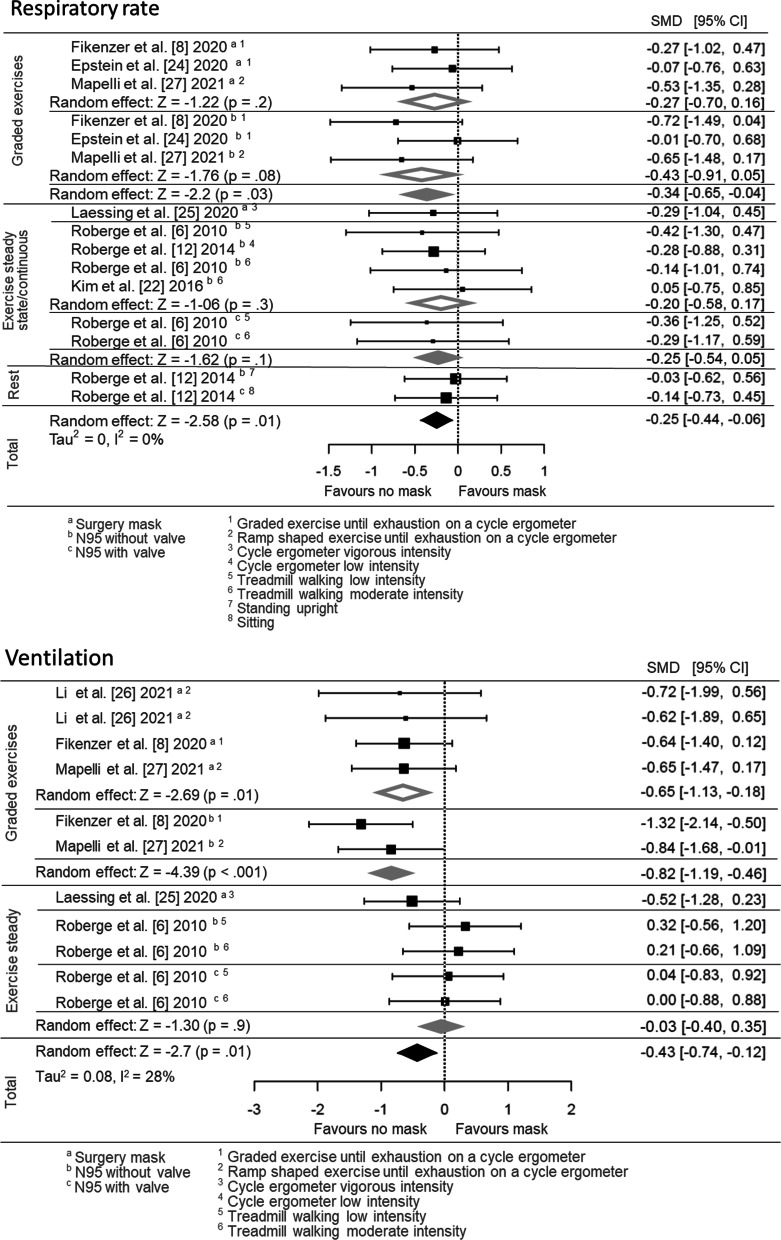
Pooled effect size estimates (standardised mean differences) for the respiratory rate and ventilation outcomes. Overall main effects for face mask application (surgery mask, FFP2/N95 with and without valve) in comparison with a comparator/no mask control, as well as the effects for subgroups based on the following grouping variables, are displayed: different mask types (surgery mask, FFP2/N95 with and without valve) and the different types of physical activity (rest, low-, moderate- and vigorous intensity). SMD, standardised mean difference; CI, confidence interval

The pooled effect estimates for pulmonary function data are displayed as forest plots in Fig. 4; both main and subgrouped (for exercise intensity and mask type) effects are shown. Mouth and nose protection using FFP2/N95 and surgical masks led to slower respiratory rates (15 effect sizes, SMD = − 0.25 [95% CI: − 0.44, − 0.06]) and to a decreased ventilation (11 effect sizes, SMD = − 0.43 [95% CI: − 0.74, − 0.12]) during physical activity and rest when compared to no mask wearing.

Four studies additionally analysed tidal volume within different exercise protocols [8, 25–27]. No effects of mask wearing during steady-state exercise occurred [25], whereas tidal volume during incremental exercise testing until volitional exertion was lower when using a surgical mask [26, 27] or FFP2/N95 respirator [8, 27] than when no mask was applied.

### Physical Performance

**Fig. 5:**
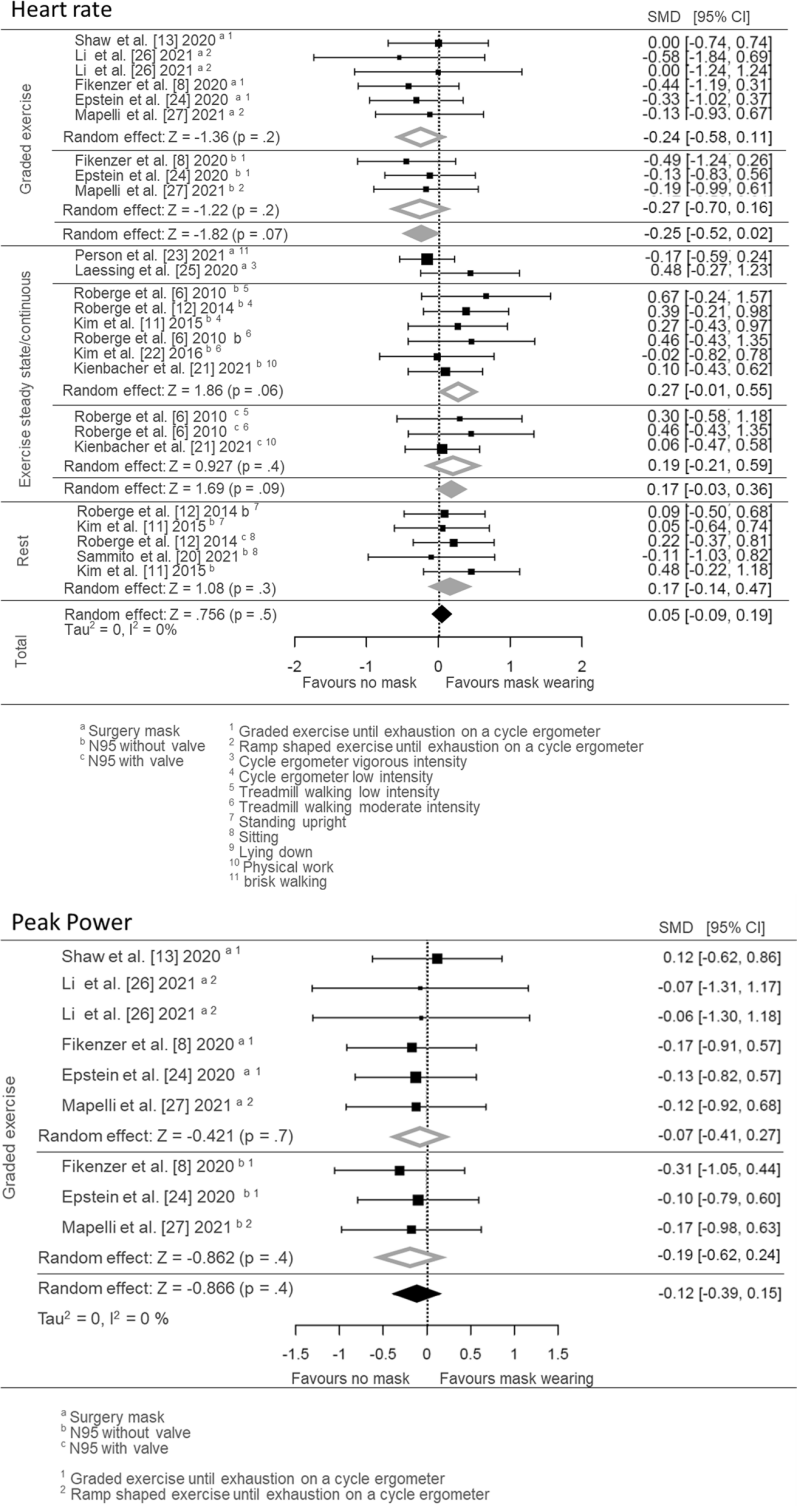
Pooled effect size estimates (standardised mean differences) for the maximal heart rate and peak power outcomes during incremental exercise testing. Overall main effects for face mask application (surgery mask, FFP2/N95 with and without valve) in comparison with a comparator/no mask control and the effects for subgroups based on the following grouping variables are shown: different mask types (surgery mask, FFP2/N95 with and without valve) and the different types of physical activity (rest, low-, moderate- and vigorous intensity). SMD, standardised mean difference; CI, confidence interval

The pooled effect estimates for heart rate and peak power output data are displayed as forest plots in Fig. 5. Both main- and subgrouped (for exercise intensity and mask type) effects were calculated. Heart rate (25 effect sizes; SMD = 0.05 [95% CI: − 0.09, 0.19]) and peak power during exercise until volitional exhaustion (9 effect sizes; SMD = − 0.12 [95% CI: − 0.39, 0.15]) were not different between mask and no mask wearing, neither in total nor dependent on the mask type (Fig. 5).

### Sensitivity Meta-regressions

The results of the sensitivity analysis on the primary outcome of pulse-derived oxygen saturation as the dependent variable are highlighted in Table 2. An impact of the participant’s age (positive, higher age leads to lower effect sizes and, thus, lower oxygen saturation during mask wearing in comparison with no mask wearing), exercise intensity (congruent to the pooled effect sizes, higher intensities lead to lower oxygen saturations during mask wearing) and mask type on the influence of mouth and nose protection (the FFP2/N95 leads to lower values than the surgery masks) on pulse-derived oxygen saturation values was found.

**Table 2:** Outcomes of the meta-regression

Mean effect size: 0.062; R^2^: 0.615; n effect sizes: 20							
Heterogeneity Q: 35.6 (df: 19, p = .01)							
	B	SE	95%CI LL	95%CI UL	Z	p value	Beta
Intercept	1.940	0.551	0.860	3.021	3.520	0.001	0.000
Age (mean) [years]	− 0.042	0.016	− 0.074	− 0.010	− 2.592	0.010	− 0.485
Exercise intensity (rest – low – moderate - vigorous)	− 0.179	0.062	− 0.299	− 0.058	− 2.905	0.004	− 0.561
Mask type (surgery – FFP2/N95 without – FFP2/N95 with valve)	− 0.271	0.133	− 0.532	− 0.009	− 2.029	0.043	− 0.381

Effect sizes, number of included effect sizes, homogeneity, the regression coefficient B, its confidence interval (CI) and the corresponding p value are displayed

### Risk of Bias Within Studies (Outcomes) and Publication Bias

**Fig. 6:**
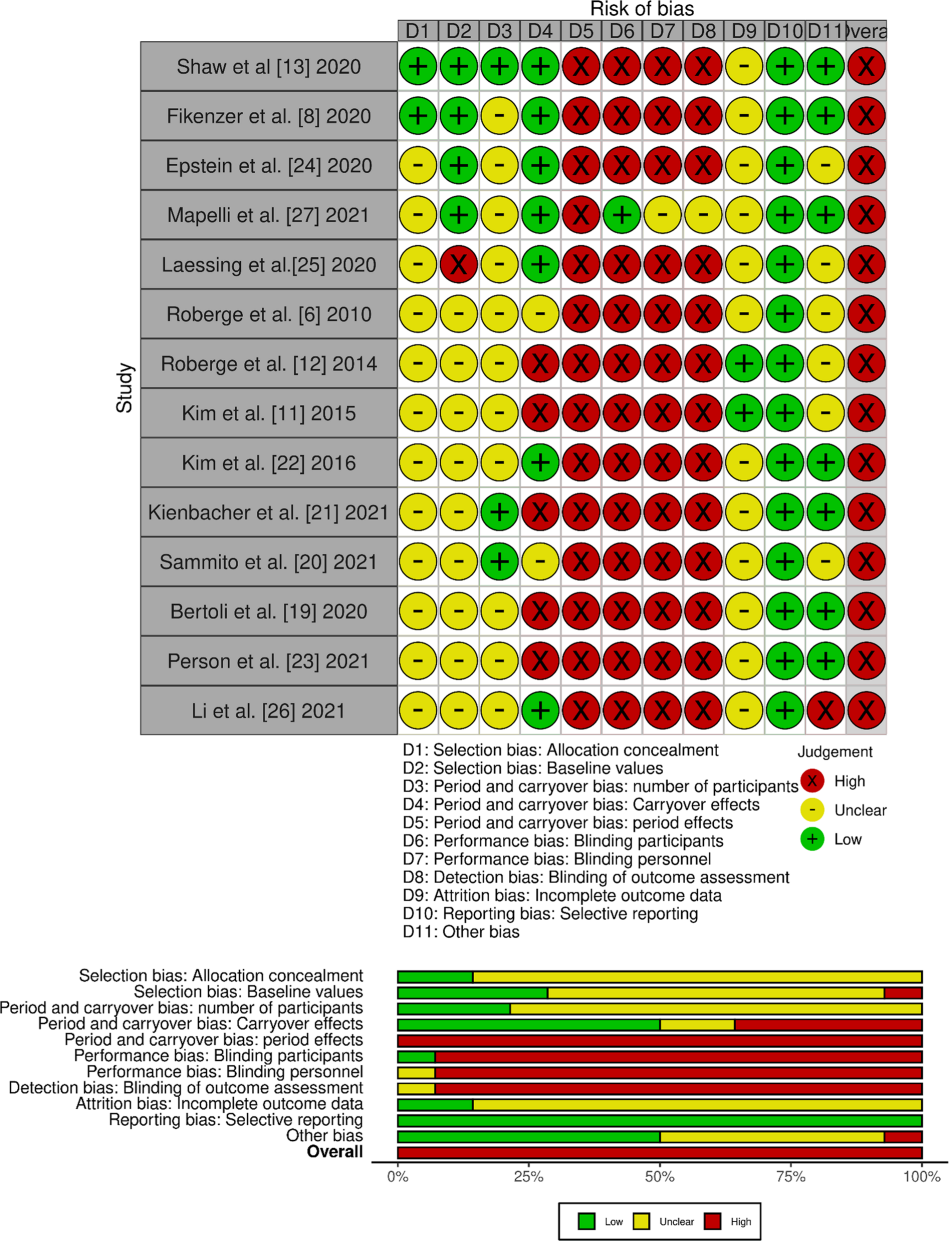
Risk of bias rating for each item, displayed as traffic light plots (above) and as summary bar plots (below). The colours indicate high (red), unclear (yellow) or low (green) risk for the respective bias domain/item

**Fig. 7:**
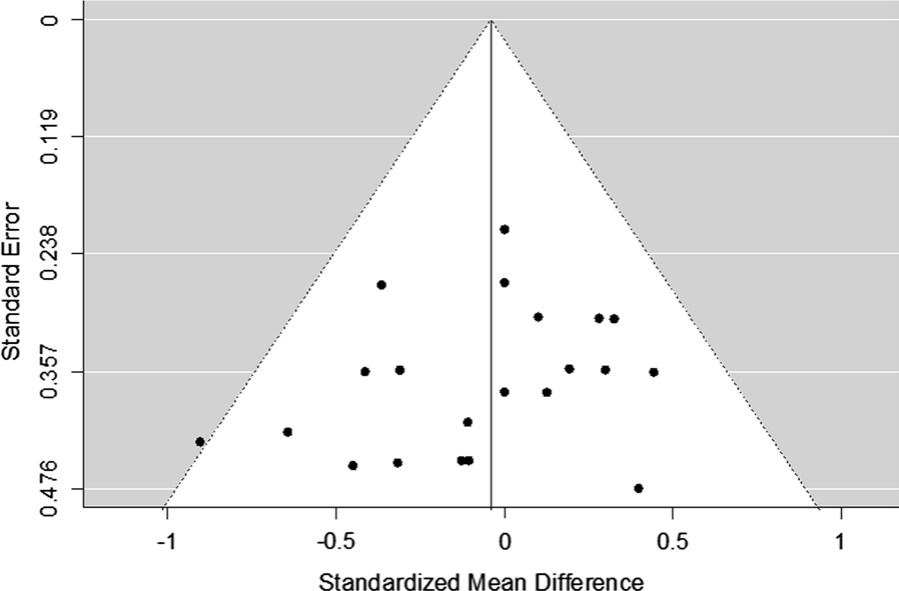
Funnel plot of all included studies. Each SMD (standard mean difference) and their corresponding SE (standard error) for pulse-derived oxygen saturation are plotted

All of the included studies showed a high overall risk of bias. Detailed ratings for the risk of bias on the study/outcome level are displayed in Fig. 6. The risk of bias across studies (publication bias) is, by means of a funnel plot, highlighted in Fig. 7. It reveals an unclear, but rather low, risk of publication bias.

## Discussion

We found low- to moderate-level evidence that the application of surgical face masks and FFP2/N95 respirators impacts gas exchange and pulmonary function. No general effect of wearing a mask on oxygen uptake was found. Subgrouping revealed that FFP2/N95 affect oxygen uptake more than surgical masks. During rest, mask wearing leads to increased oxygen saturation, whereas exhausting physical activity with a mask led to decreases in oxygen uptake and saturation compared to exercise without a mask. Although these effects indicate that aerobic capacity is negatively affected by mask wearing, peak performance was comparable with and without mouth and nose protection.

Ventilation and respiratory rates are lowered during rest and physical activity when a medical mask is worn, irrespective of the mask type. In contrast, carbon dioxide metabolism seems to be compensated, even during strenuous exercise.

On first sight, the finding that oxygen saturation is increased during rest when a mask is worn is somewhat surprising. To interpret the results further, it is crucial to keep in mind that, during rest, carbon dioxide is most likely the primary stimulus for respiratory drive and that adjustments to breathing patterns include changes in ventilation, respiratory rate and tidal volume [29]. As a potential explanation, carbon dioxide concentrations of up to 3% in the dead space between the face and a filtering face piece respirator (compared to 0.04% carbon dioxide in ambient air) were described [30]. If this, previously exhaled air, is trapped within the mask and re-inhaled during mask application, higher concentrations of carbon dioxide within the alveolar air result [4]. Since the respiratory rate and ventilation were negatively affected by increased breathing resistance, an increase in tidal volume is a plausible response to increased respiratory drive during rest [5]. In line with this, a documented decrement in respiratory rate is discussed as a potential effect of increased tidal volume [12]. We assume that the increased tidal volume during rest may be sufficient to counteract the influence of the increased dead space volume and breathing resistance on carbon dioxide exhalation. As a side effect of higher inspiratory volume per breath, alveolar oxygen uptake and, thus, oxygen saturation are increased during rest. Since none of the included studies assessed tidal volume during rest or arterial oxygen partial pressure, future studies are needed to confirm or falsify this hypothesis.

A diverging effect on oxygen uptake and pulse-derived oxygen saturation was reported for mask application during exercise with vigorous intensity. Early physiological studies found a positive correlation between the intensity of physical activity and the impact of increased breathing resistance on decreased ventilation and respiratory rates [5]. Tidal volume, on the other hand, seems to increase only during rest and light activity and tends to decrease during higher intensity exercise when breathing resistance is elevated [5]. Three of the included studies confirm lower tidal volumes during exhausting exercise when surgical masks [26, 27] or FFP2/N95 respirators [8, 27] are worn; these findings were accompanied by lower respiratory rates and ventilation. These limitations in all three markers of pulmonary function (tidal volume, respiratory rate and ventilation) limit the oxygen uptake response to exercise when a mask is worn [31] and, thus, lead to significantly lower oxygen saturation as indicated by our meta-analysis. It is, however, unlikely that these alterations in oxygen saturation lead to clinical symptoms. Although being lower, compared to a no mask comparator, tidal volume, ventilation and respiratory rate increase during exercise compared to the resting state. Therefore, the proportion of re-inhaled air compared to overall tidal volume is considerably lower during exercise than during rest. In line with this hypothesis, a metabolic simulation reports a lower impact on inhaled carbon dioxide concentrations (< 2% carbon dioxide concentration) and higher inhalation and exhalation pressures during activities with high intensity compared to activities with low intensity when a FFP2/N95 respirator is applied [4]. This may provide an explanation for the lack of detrimental changes in carbon dioxide exhalation during exhausting exercise reported by Mapelli and colleagues [27]. A larger body of evidence, including information on carbon dioxide partial pressure and anaerobic metabolism, is necessary to confirm that carbon dioxide metabolism is not impaired during physical activity when a surgical mask or FFP2/N95 respirator is applied [32].

The pooled effects for both medical mask types indicated that mask application during rest and physical activity increases breathing resistance and, thus, affects respiration. In line with our hypothesis, effects on oxygen saturation were related to mask type, indicating a greater impact of FFP2/N95 respirators compared to surgical masks. One explanation, therefore, is that the tighter fit of the FFP/N95 respirators provides a better leakage sealant than the surgical mask. Based on the small number of randomised controlled trials, we are not able to differentiate further the effects of mask types on respiration and aerobic metabolism. Although respirators with exhalation valves are reported to be more comfortable [30], we found no further differences between the effect of respirators with and without exhalation valves on gas exchange or respiratory function.

### Limitations

None of the studies available assessed the effect of mask applications over time frames larger than 60 min or the impact of repeated mask application in real-life situations during the day. Most of the studies applying spiroergometry [8, 26, 27] had methodological issues leading to a high risk of bias. Both surgical masks and FFP2/N95 respirators were worn under a rubber mask (to measure breathing components) which may have affected the surface for gas exchange, the sealant and dead space between the face and respirator and breathing resistance (although the rubber mask was, of course, also worn during the no mask conditions). Furthermore, the application of rubber masks over FFP2/N95 and surgical masks might have led to greater leakage of gas and therefore to changes in gas exchange values [33].

Only three studies applied invasive assessments for oxygen and carbon dioxide measurements and no study, so far, has analysed arterial oxygen saturation rather than capillary measurements. These limitations must also be considered when our findings are interpreted.

The studies included show a high individual risk of bias. On the contrary, the risk of bias across studies (publication bias) seems to be low. As only 14 RCTs were available to be analysed, following our rigorous inclusion and exclusion criteria, the number of included studies was quite small. Furthermore, most of the studies did not compare multiple mask types within the same design. As a result, the evidence concerning the impact of mask types or physical activity characteristics is only preliminary.

### Practical Relevance

Our data confirm that healthy adults can compensate the impact of mouth and nose protection during rest and (most) physical activities up to moderate intensity. We can, therefore, confirm the assumption that mask wearing does not induce clinically relevant hypoxia or hypercapnia if a metabolic steady state can be obtained. Although self-report complaints, including headache or impaired cognitive performance, could be associated with slightly elevated carbon dioxide concentrations [9], it is more likely that discomfort during mask wearing [8] and respiratory fatigue [4] may account for impaired work capacity and premature fatigue. In view of the current international recommendations, the application of surgical masks seems to have a better risk–benefit-balance than the application of FFP2/N95 respirators during rest and physical work, provided that FFP2/N95 respirator application has no advantage concerning the prevention of airborne virus transmission [3].

## Conclusions

Exhausting high intensity activities seem to induce a lower oxygen uptake and availability (in particular with the FFP2/N95 respirator) when a mask is applied. It is likely that decreased oxygen uptake capacity leads to a higher proportion of anaerobic metabolism during exhausting exercise with a comparable workload. Against earlier assumptions, we could not confirm a detrimental effect on maximal performance [10]. Since perceived exertion during exercise was reported to be higher during exercise with masks [13], more randomised controlled studies applying exercise of vigorous intensity with a matched workload are needed to confirm that endurance is not limited. Since detrimental effects cannot be ruled out, exhausting physical activity with a mask cannot unconditionally be encouraged based on the pooled data of this review. This recommendation may be of higher relevance for occupational settings than for leisure time exercise which, currently, should take place outdoors.

## Data Availability

The data set for meta-analytic calculations will be made available via direct contact with investigator and after approval of a written proposal and a signed data access agreement.

## Abbreviations

FFP2: Filtering face piece type 2
N95: Face piece type N95
COVID-19: Corona virus disease 2019
SpO_2_: Pulse-derived oxygen saturation
PCO_2_: Carbon dioxide partial pressure
VCO_2_: Carbon dioxide exhalation
VO_2_: Oxygen uptake
HR: Heart rate
W_peak_: Peak power output
RCT: Randomised controlled trial
SMD: Standardised mean differences
CI: Confidence interval
WHO: World Health Organization
CO_2_: Carbon dioxide
O_2_: Oxygen
PRISMA: Preferred Reporting Items for Systematic Reviews and Meta-Analyses
CPET: Cardiopulmonary exercise testing
RoB II: Revised Cochrane risk of bias tool
SD: Standard deviation
n: Number
n.a.: Not applicable
RPE: Rating of perceived exertion
min: Minutes
kg: Kilogrammes
cm: Centimetres
CI: Confidence interval
SMD: Standardised mean difference
